# Crohn’s disease-specific mortality: a 30-year cohort study at a tertiary referral center in Japan

**DOI:** 10.1007/s00535-018-1482-y

**Published:** 2018-06-09

**Authors:** Shigeyoshi Yasukawa, Toshiyuki Matsui, Yutaka Yano, Yuho Sato, Yasumichi Takada, Masahiro Kishi, Yoichiro Ono, Noritaka Takatsu, Takashi Nagahama, Takashi Hisabe, Fumihito Hirai, Kenshi Yao, Toshiharu Ueki, Daijiro Higashi, Kitaro Futami, Suketo Sou, Toshihiro Sakurai, Tsuneyoshi Yao, Hiroshi Tanabe, Akinori Iwashita, Masakazu Washio

**Affiliations:** 1 Department of Gastroenterology, Fukuoka University Chikushi Hospital, 1-1-1 Zokumyoin, Chikushino, Fukuoka 818-0067 Japan; 2 Inflammatory Bowel Disease Center, Fukuoka University Chikushi Hospital, Fukuoka, Japan; 3 Department of Endoscopy, Fukuoka University Chikushi Hospital, Fukuoka, Japan; 4 Department of Surgery, Fukuoka University Chikushi Hospital, Fukuoka, Japan; 5 Department of Gastroenterology, Tobata Kyoritsu Hospital, Kitakyushu, Japan; 6 Department of Gastroenterology, Ashiya Central Hospital, Kitakyushu, Japan; 7 Department of Gastroenterology, Sada Hospital, Fukuoka, Japan; 8 Department of Pathology, Fukuoka University Chikushi Hospital, Fukuoka, Japan; 9 Department of Community Health and Clinical Epidemiology, St. Mary’s College, Kurume, Japan

**Keywords:** Crohn’s disease-specific mortality, Standardized mortality ratios, Cohort study, Intestinal cancer, Amyloidosis

## Abstract

**Background:**

In this study, survival and cause of death were investigated in patients with Crohn’s disease (CD) at a tertiary referral center.

**Methods:**

A database was created based on the medical records of 1108 CD patients who had a history of visiting our hospital to investigate background characteristics, cumulative survival rates from diagnosis, causes of death, and the standardized mortality ratio (SMR) for each cause of death. A follow-up questionnaire survey of patients followed up inadequately was also conducted. The cumulative survival rate from diagnosis was determined using the life table method and compared with that of a sex- and age-matched population model from the year 2000.

**Results:**

The study included 1108 patients whose mean age at diagnosis was 25.6 ± 10.8 years. The mean duration of follow-up was 14.6 ± 9.4 years, and there were 52 deaths. The cumulative survival rate was significantly lower 25 years after the diagnosis of CD (91.7%) than in the standard population model (95.7%). SMRs for both all causes [3.5; 95% confidence interval (CI): 2.7–4.6] and CD-specific causes (36.7; 95% CI 26.1–51.6) were high. Among the CD-specific causes, SMRs were especially high for small intestine and colorectal cancers, gastrointestinal diseases including intestinal failure (IF), perioperative complications, and amyloidosis.

**Conclusion:**

The SMRs for both all causes and CD-specific causes were high in CD patients. CD-specific causes including intestinal cancer, IF, perioperative complications, and amyloidosis showed especially high SMRs.

## Introduction

Crohn’s disease (CD) is a chronic inflammatory bowel disease (IBD) of unknown etiology. It often develops at an early age and can affect the entire gastrointestinal tract from the oral cavity to the anus. It is also known to frequently require intestinal resection due to the development of intestinal complications such as stenosis and fistula through repeated relapses or recurrences [1–4]. Although there are some reports of the long-term course of patients with this disease, there are very few reports related to their survival and cause of death; moreover, most previous reports are derived from population-based cohort studies, and there are very few, long-term, hospital-based, cohort studies with a long duration of follow-up and a large sample size [5–14]. In the present study, a database based on the medical records of CD patients at our hospital with the addition of information obtained from a questionnaire survey was created, and the long-term prognosis of CD patients was investigated.

## Methods

### Study design and populations

This was a single-center, retrospective, cohort study conducted at Fukuoka University Chikushi Hospital. Among the patients with a definitive diagnosis of CD who had a history of visiting our hospital between May 1967 and December 2015, patients with a history of treatment for at least 6 months since diagnosis were selected, and a database based on their medical records was subsequently created [3]. Patients who were followed up for less than 6 months after diagnosis were excluded. First, sex, age at diagnosis, date of diagnosis, smoking history, disease type at diagnosis, presence/absence of perianal disease, survival to the last observed day, surgical history, and use of medications (aminosalicylates, corticosteroids, thiopurines, and anti-tumor necrosis factor [TNF] agents) were determined from the medical records. Subsequently, a questionnaire survey of the patients who had not visited our hospital for at least 3 years was conducted between May 2015 and May 2016.

In the present study, (1) the cumulative mortality of CD patients was compared with that of the standard population, (2) the causes of death in CD and the standardized mortality ratios (SMRs) by cause of death were investigated, and (3) background characteristics that affect mortality in CD patients were investigated.

### Duration of follow-up

Duration of follow-up was defined as from the date of diagnosis to the last observed day. The date of diagnosis was determined based on medical records at our hospital, and the last observed day was the date of the final visit to our hospital according to the medical records, or the date of survey response or date of death for patients whose information could be collected through the follow-up questionnaire survey.

### Ever-use of medicine

The use of medications was reviewed for the duration of follow-up in all patients, and the use of aminosalicylates, corticosteroids, thiopurines, and anti-TNF agents was examined. Duration of medication use, dose, and side effects were not taken into consideration.

### Diagnosis and treatment of CD

CD was diagnosed based on clinical symptoms, endoscopic findings, X-ray findings, and histological findings in accordance with the diagnostic criteria for CD proposed by the Japanese Ministry of Health, Labour and Welfare [15–17]. Furthermore, using the Montreal classification, disease location at diagnosis was classified into ileal type, colonic type, or ileocolonic type, and disease behavior at diagnosis was classified into inflammatory, stricturing, or penetrating type [18].

### Assessment of mortality

Date and cause of death were determined based on medical records, and for the patients whose deaths were identified through the follow-up questionnaire survey were determined through an inquiry to the facility where they passed away. As in previous reports, the causes of death were classified in accordance with the *International Classification of Diseases 10th revision* (*ICD*-*10*) [13, 14, 19].

Gastrointestinal disease (excluding non-alcoholic liver disease, ICD codes K00–K70, 77–93) included intestinal complications of CD such as intestinal obstruction and perforation, perioperative complications, state of undernutrition due to short-bowel syndrome, and sudden death of unknown cause that occurred during home parenteral nutrition (HPN) or the active stage of the disease. Non-alcoholic liver disease (ICD code: K71–76) included chronic and progressive liver diseases that were not considered associated with CD.

There are no previous reports of the SMRs for deaths related to CD. Therefore, with the aim to calculate CD-specific mortality, the total mortality for small intestinal cancer (ICD code: C17), colorectal cancer (CRC) (ICD code: C18–21), and amyloidosis (ICD code: E85), as well as gastrointestinal diseases, as CD-specific causes was determined, and the numbers of expected cases of death and SMRs were calculated from the above mortality and person-years in the sex- and age-matched population model.

### Statistical analysis

1. Expected and observed cumulative survival rates from diagnosis
  The age by sex-specific death rates of the Japanese population in 2000 were applied to the distribution of the age groups of the study population by sex to estimate the yearly expected survival rate of the general population during the follow-up period. The death rates for 5-year age bands of both sexes were used in this study. According to the past reports, the age by sex-specific mortality of the Japanese population in 2000 [20], which was almost the median of the year of diagnosis, was used [13]. The life table method was used to estimate the cumulative survival rate and 95% confidence intervals of the study subjects.
2. SMRs of patients with CD
  The SMR is the ratio of the observed number of deaths among the patients with CD in this cohort study to the expected number of deaths in the study population under the assumption that the age by sex-specific mortality rates for the study population are the same as those for a reference population. The expected number of deaths was calculated by multiplying the person-years of the study population by the age by sex-specific death rates of the Japanese population in 2000. The SMRs were calculated for 5-year age bands of both sexes in this study.
3. Factors associated with mortality of patients with CD
  Hazard ratios of death and their 95% confidence intervals were estimated with a Cox proportional hazards model. Age was treated as a continuous variable, while other factors were treated as indicator variables.
4. Number of intestinal resections and mortality in patients with CD.
  Number of intestinal resection was classified into three categories: 0, 1–4, 5 or more. A Chi-squared test was used to evaluate if the number of intestinal resections was related to the death rate. The dose-dependent trend was tested by evaluating the regression coefficient when the categories were treated as equally spaced numerical variables in Cox’s model

Person-years at risk were computed from the date of CD diagnosis until the date of death, loss to follow-up, or end of follow-up.

All statistical analyses were conducted using the Statistical Analysis System (SAS Institute, Cary, NC, USA) package. A two-sided *P* less than 0.05 was considered significant.

### Ethical considerations

This study was conducted in accordance with the 1964 Helsinki Declaration and its subsequent amendments. Personal information listed in the medical records or obtained through questionnaire surveys, including informed consent, was managed on a database such that individuals were anonymized. This study was approved by the institutional review board of Fukuoka University Chikushi Hospital (R14-011, 2014.6.4–2017.2.28).

## Results

### Patient populations and background characteristics

This study included 1108 patients whose duration of follow-up was at least 6 months from among the 1165 CD patients who were diagnosed after May 1967 and had a history of visiting our hospital to December 2015. A questionnaire survey was distributed to 303 CD patients who did not have a history of visiting our hospital for at least 3 years, and information on their present condition (alive, dead), cause of death, last observed day, smoking history, surgical history, and previously used medications was added from the 106 responses. It was, therefore, possible to obtain information on background characteristics and survival to the last observed day for all patients. The patients’ clinical characteristics are shown in Table 1. There were 758 men and 350 women who were 7–76 years old at the time of diagnosis. The mean age at diagnosis was 25.6 ± 10.8 years, and the mean duration of follow-up was 14.6 ± 9.4 years (range 0.5–47.2 years). The study included 16,199 person-years, and 583 patients (52.6%) were diagnosed before 2000. Disease location at diagnosis was ileal type in 386 patients (34.8%), colonic type in 197 patients (17.8%), and ileocolonic type in 525 patients (47.4%). Disease behavior was inflammatory in 531 patients (47.9%), stricturing in 365 patients (32.9%), penetrating in 208 patients (18.8%), and unknown in 4 patients (0.4%). A perianal fistula was present at diagnosis in 594 patients (53.6%). There were 303 current smokers (27.3%), 610 never-smokers (55.1%), and 117 ex-smokers (10.6%).

**Table 1:** Characteristics of patients with Crohn’s disease (*n* = 1108)

Sex (male/female)	758/350
Age at diagnosis (mean ± SD, years)	25.6 ± 10.8 (7–76)
Follow-up period (mean ± SD, years)	14.6 ± 9.4 (0.5–47.2)
Calendar year at diagnosis	
< 2000	583 (52.6%)
≥ 2000	525 (47.4%)
Disease location at diagnosis^a^	
Ileal	386 (34.8%)
Colonic	197 (17.8%)
Ileocolonic	525 (47.4%)
Disease behavior at diagnosis^a^	
Inflammatory	531 (47.9%)
Stricturing	365 (32.9%)
Penetrating	208 (18.8%)
Unknown	4 (0.4%)
Perianal disease at diagnosis	
Absent	514 (46.4%)
Present	594 (53.6%)
Smoking at last observed day	
Never-smoker	610 (55.1%)
Ex-smoker	117 (10.6%)
Current smoker	303 (27.3%)
Unknown	78 (7.0%)
Intestinal resection at last observed day	
Absent	446 (40.3%)
Present	662 (59.7%)
Number of intestinal resections (mean ± SD)	1.2 ± 1.4 (0–7)
Ever-use of medication	
Aminosalicylates	864 (77.9%)
Corticosteroids	346 (31.2%)
Thiopurines	423 (38.1%)
Anti-TNF agents	570 (51.4%)

*SD* standard deviation, *TNF* tumor necrosis factor

^a^Disease location and behavior at diagnosis are according to the Montreal classification

There were 662 patients (59.7%) who had undergone intestinal surgery by the last observed day. The mean number of surgeries per patient was 1.2 ± 1.4. Previously used medications included aminosalicylates in 864 patients (77.9%), corticosteroids in 346 patients (31.2%), thiopurines in 423 patients (38.1%), and anti-TNF agents in 570 patients (51.4%).

### Overall mortality

Fifty-two deaths occurred among the 1108 patients with CD. Among the 52 deaths, 9 were reported through the additional questionnaire survey. The background characteristics of the deceased patients are shown in Table 2. Of the 52 patients who died, 32 were men (61.5%). The mean age at diagnosis was 29.1 ± 14.0 years, the mean age at death was 48.1 ± 14.6 years, and the mean duration of follow-up from diagnosis to death was 18.3 ± 8.8 years. Disease location at diagnosis was ileal type in 15 patients (28.9%), colonic type in 6 patients (11.5%), and ileocolonic type in 31 patients (59.6%). Disease behavior at diagnosis was inflammatory in 21 patients (40.4%), stricturing in 19 patients (36.5%), and penetrating in 12 patients (23.1%). There were 31 patients (59.6%) presenting with perianal disease, 2 current smokers (3.9%), and 45 patients (86.5%) who had undergone surgery during the follow-up period. The mean number of surgeries per patient was 2.3 ± 1.8. Previously used medications were aminosalicylates in 29 patients (55.8%), corticosteroids in 26 patients (50.0%), thiopurines in 12 patients (23.1%), and anti-TNF agents in 21 patients (40.4%).

**Table 2:** Background characteristics of deceased patients (*n* = 52)

Sex (male)	32 (61.5%)
Age at diagnosis (mean ± SD, years)	29.1 ± 14.0
Age at death (mean ± SD, years)	48.1 ± 14.6
Duration of follow-up (mean ± SD, years)	18.3 ± 8.8
Disease location at diagnosis^a^	
Ileal	15 (28.9%)
Colonic	6 (11.5%)
Ileocolonic	31 (59.6%)
Disease behavior at diagnosis^a^	
Inflammatory	21 (40.4%)
Stricturing	19 (36.5%)
Penetrating	12 (23.1%)
Perianal disease at diagnosis	31 (59.6%)
Smoking at last observed day	
Never-smoker	38 (73.1%)
Ex-smoker	11 (21.1%)
Current smoker	2 (3.9%)
Unknown	1 (1.9%)
Intestinal resection at last observed day	
Present	45 (86.5%)
Number of intestinal resections (mean ± SD)	2.3 ± 1.8 (0-7)
Ever-use of medication	
Aminosalicylates	29 (55.8%)
Corticosteroids	26 (50.0%)
Thiopurines	12 (23.1%)
Anti-TNF agents	21 (40.4%)

*SD* standard deviation, *TNF* tumor necrosis factor

^a^Disease location and behavior at diagnosis are according to the Montreal classification

### Expected and observed cumulative survival rates from diagnosis

**Fig. 1:**
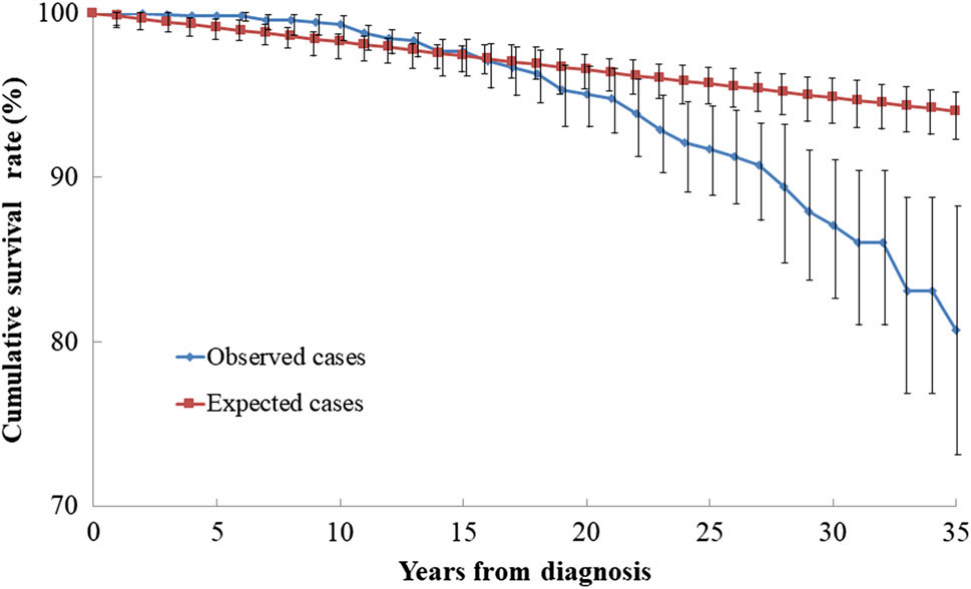
Expected and observed cumulative survival rates from diagnosis in 1108 patients with CD. Although the cumulative survival rate at 25 years from diagnosis is not different compared to that of the standard population model, it is significantly lower ≥ 25 years after diagnosis

The cumulative survival rates from diagnosis in CD patients are shown in Fig. 1. The cumulative survival rates among CD patients were 99.3% (95% CI 98.8–99.9) after 10 years (expected cumulative survival rate: 98.3%, 95% CI 97.5–99.0%), 95.1% (95% CI 93.3–96.9%) after 20 years (expected cumulative survival rate: 96.5%, 95% CI 95.5–97.6%), and 87.1% (95% CI 82.8–91.4%) after 30 years (expected cumulative survival rate: 94.9%, 95% CI 93.6–96.1%). The cumulative survival rate of CD patients in the first 24 years since diagnosis was not different from that of the standard population model. However, it was significantly lower in CD patients (91.7%; 95% CI 89.0–94.4%) at 25 years after diagnosis compared to that in the standard population model (95.7%; 95% CI 94.5–96.9%), and this pattern continued thereafter.

### Causes of death in CD and the SMRs by cause of death

The numbers of deaths and SMRs by cause of death in CD patients are shown in Table 3. Fifty-two of the 1108 CD patients died. The causes of death were: malignant tumor (*n* = 19; small intestinal cancer, *n* = 2; CRC, *n* = 11; lung cancer, *n* = 2; lymphoma, *n* = 2; brain tumor, *n* = 1; and cancer of unknown primary, *n* = 1), gastrointestinal disease (*n* = 15), liver disease (*n* = 3), respiratory disease (*n* = 2), cardiovascular disease (*n* = 3), suicide (*n* = 3), metabolic disease (*n* = 5), and traffic injuries (*n* = 1). Information about the cause of death was not provided on the questionnaire survey in one case. There were a total of 33 deaths related to CD, including small intestinal cancer, CRC, gastrointestinal disease, and amyloidosis (pathologically secondary amyloidosis as shown by immunostaining AA amyloid). Gastrointestinal disease (*n* = 15) consisted of intestinal failure (IF) (*n* = 7), severe (fistulous) disease including perioperative complications (*n* = 6), and intestinal perforation (*n* = 2). Amyloidosis (*n* = 5) consisted of kidney failure (*n* = 3) and massive bleeding (*n* = 2).

**Table 3:** All causes of death in Crohn’s disease patients and standardized mortality ratios by cause of death

Cause	ICD-10 code	Observed cases	Expected cases	SMR	95% CI
All causes		52	14.8	3.5	2.7–4.6
Malignant tumor	C00–D48	19	3.5	5.4	3.5–8.5
Small intestinal cancer^a^	C17	2	0.01	200	500.1–7997.1
Colorectal cancer^a^	C18–C21	11	0.37	29.7	16.5–53.7
Lung cancer	C33–C34	2	0.45	4.4	1.1–17.8
Lymphatic cancer	C81–C96	2	0.72	2.8	0.7–11.1
Brain tumor	C70–C72	1	0.08	12.5	1.8–88.7
Cancer of unknown primary	–	1	–	–	–
Gastrointestinal disease^a^ (excluding K71–76)	K00–70,77–93	15	0.31	48.4	29.2–80.3
Non-alcoholic liver disease	K71–76	3	0.21	14.3	4.6–44.3
Respiratory disease	J00–J99	2	1.4	1.4	0.4–5.7
Cardiovascular disease	I00–I99	3	2.4	1.3	0.4–3.9
Suicide	X60–X84	3	2.6	1.2	0.4–3.6
Metabolic disease (amyloidosis)^a^	E85	5	0.005	1000	416.2–2402.6
Traffic injuries	V00–V89	1	1.4	0.7	0.1–5.1
Unknown	–	1	–	–	–
CD-specific causes^a^		33	0.9	36.7	26.1–51.6

*ICD-10* International Classification of Diseases-10, *SMR* standardized mortality ratio, *CI* confidence interval

^a^CD-specific causes are small bowel and colorectal cancer, gastrointestinal disease, and amyloidosis

### SMRs

Fifty-two all-cause deaths were observed in this study, while the expected value calculated from the mortality in the sex- and age-matched general population was 14.8. Thus, the SMR for all-cause mortality was 3.5 (95% CI 2.7–4.6).

Analysis by disease revealed a high SMR of 5.4 (95% CI 3.5–8.5) for malignant tumors. SMRs were especially high for small intestinal cancer at 200 (95% CI 500.1–7997.1), CRC at 29.7 (95% CI 16.5–53.7), and brain tumor at 12.5 (95% CI 1.8–88.7). In addition, SMRs were also high for gastrointestinal disease at 48.4 (95% CI 29.2–80.3), non-alcoholic liver disease at 14.3 (95% CI 4.6–44.3), and amyloidosis at 1000 (95% CI 416.2–2402.6). However, high SMRs were not observed for many conditions, such as respiratory disease (1.4; 95% CI 0.4–5.7), cardiovascular disease (1.3; 95% CI 0.4–3.9), suicide (1.2; 95% CI 0.4–3.6), and traffic injuries (0.7; 95% CI 500.1–7997.1). The SMR for CD-specific causes of death, which included small intestinal cancer, CRC, gastrointestinal disease, and metabolic disease, was high at 36.7 (95% CI 26.1–51.6).

Thus, the results showed that many deaths of CD patients were caused by intestinal cancer, IF, severe (fistulous) disease including perioperative complications, and amyloidosis.

### Factors associated with mortality in patients with CD

Factors associated with mortality are shown in Table 4. Sex, calendar year, disease location and behavior at diagnosis, smoking habits, perianal fistula, thiopurines, and use of anti-TNF agents were not significant factors. However, old age at diagnosis (≥ 60 years) [hazard ratio (HR) 19.93; 95% CI 5.56–71.54] and use of corticosteroids (HR 1.94; 95% CI 1.12–3.25) had high HRs.

**Table 4:** Factors associated with mortality of Crohn’s disease patients

Data at the baseline survey	Person-years	No. of cases	Age- and sex-adjusted HR (95% CI)
Sex			
Female	5438	20	1.00 (reference)
Male	10,759	32	0.97 (0.55–1.07)
Age (years)			
< 20	5549	14	0.64 (0.34–1.19)
20–59	10,505	35	1.00 (reference)
≥ 60	142	3	19.93 (5.56–71.54)
Calendar year			
< 1999	12,126	47	1.00 (reference)
≥ 2000	4071	5	0.61 (0.21–1.74)
Disease location at diagnosis			
Ileal	5859	15	0.57 (0.31–1.05)
Colonic+ileocolonic	10,338	37	1.00 (reference)
Disease behavior at diagnosis			
Inflammatory	6795	15	1.03 (0.59–1.80)
Stricturing + penetrating	9335	37	1.00 (reference)
Perianal disease			
Absent	6558	21	1.00 (reference)
Present	9639	31	1.07 (0.60–1.90)
Smoking habits			
Never-smoker + ex-smoker	10,619	40	1.00 (reference)
Current smoker	4649	11	0.77 (0.38–1.52)
Intestinal resection			
Absent	4273	7	1.00 (reference)
Present	11,925	45	1.47 (0.65–3.35)
Ever-use of medicine corticosteroids			
Without	10,502	26	1.00 (reference)
With	5509	26	1.94 (1.12–3.25)
Thiopurines			
Without	9868	40	1.00 (reference)
With	6184	12	0.59 (0.30–1.13)
Anti-TNF agents			
Without	7945	31	1.00 (reference)
With	8253	21	0.87 (0.49–1.55)

HR, hazard ratio; CI, confidence interval; TNF, tumor necrosis factor

### Number of intestinal resections and the death rate

The number of intestinal resections and the death rate in all subjects and in patients followed up more than 25 years were examined (Table 5). A higher number of surgeries was significantly associated with higher mortality during the entire follow-up period (Table 5, *p* < 0.01 by Chi-squared test, *p* for trend < 0.01), as well as in patients who were followed up for 25 years and more (Table 5, *p* = 0.03 by Chi-squared test, *p* for trend = 0.02).

**Table 5:** Number of intestinal resections and death rates by follow-up period

Present condition	Number of intestinal resections			*p* for trend**
	0 (*n* = 446)	1–4 (*n* = 620)	≥ 5 (*n* = 42)	
(a) Death rate and number of intestinal resections in all subjects (total observation period)				
Dead (*n* = 52)	7 (1.6%)	36 (5.8%)*	9 (21.4%)*	<0.01

Present condition	Number of intestinal resections			*p* for trend**
	0 (*n* = 24)	1–4 (*n* = 137)	≥ 5 (*n* = 23)	
(b) Death rate and number of intestinal resections in patients observed more than 25 years				
Dead (*n* = 12)	0 (0.0%)	8 (5.8%)	4 (17.4%)*	0.02

Data were presented as *n* (%)

*p < 0.03 vs no intestinal resection * Chi-squared test

**Regression coefficient using Cox’s model

## Discussion

The results of long-term follow-up of CD patients in the present study showed that the cumulative survival rate decreased and SMR increased ≥ 25 years after diagnosis. Among individual complications, CD-specific causes, such as intestinal cancer, IF, severe (fistulous) diseases including perioperative complications, and amyloidosis, developed ≥ 20 years after diagnosis, increasing mortality.

There are several reports primarily from Western countries concerning the survival and causes of death of CD patients. It has recently been reported that mortality is similar or slightly higher in CD patients than in the general population [5–14]. However, most data are derived from population-based studies, which are considered not conducive to patient follow-up or detailed investigation of the causes of deaths, and there are very few, long-term, hospital-based studies with a large sample size [5, 6, 14, 21–25]. Therefore, this hospital-based cohort study was conducted at a tertiary referral center in Japan to investigate survival, cause of death, and factors that affect mortality of CD patients in detail.

The cumulative survival rates of CD patients from diagnosis were 98.9% at 10 years, 94.0% at 20 years, and 86.7% at 30 years according to Lee et al. from Korea [14], and the rates were generally consistent with the results of the present study. Some reports compared the cumulative survival rate from diagnosis with that of the standard population model and found no significant differences between the two groups [5, 8, 11]. Consistent with previous reports, the present study results also did not show significant differences for 25 years from diagnosis. However, longer follow-up demonstrated that the cumulative survival rate decreased significantly ≥ 25 years from diagnosis. These findings suggest that long-term follow-up of 20 years or longer may show a decrease in cumulative survival among CD patients.

Table 6 shows SMRs for all causes from previous hospital-based studies [5, 6, 14, 21–25]. Prior et al. [21] and Weterman et al. [22] reported high SMRs, but recent reports did not find increased SMRs. Except for the study by Lee et al., previous reports encompassed a low number of person-years (range 2712–7424), which takes into account the number of patients and the duration of follow-up. Furthermore, compared to the report by Lee at al., the present study had a longer mean duration of follow-up (14.6 years) with 16,197 person-years. It is evident from Fig. 1 that the cumulative survival rate > 25 years after diagnosis was decreased, indicating that the present study might be informative, since there are no other studies with both high person-years at risk and a long duration of follow-up.

**Table 6:** Hospital-based studies examining mortality in Crohn’s disease

Author	Year	Country	Years of diagnosis	Follow-up period (years)	Cases	Deaths	SMR	95% CI	%Death	Person-years at risk
Prior	1981	UK	1941–1976	14.5	513	102	2.0	1.85–2.47	19.9	7424
Weterman	1990	Netherlands	1934–1984	9.9	659	64	2.2	1.75–2.85	9.7	6590^a^
Cottone	1996	Italy	1973–1993	7.8	531	9	1.0	0.4–1.8	1.7	2712
Farrokhyar	2001	UK	1978–1986	15.0	196	23	0.9	0.59–1.4	11.7	3623
Uno	2003	Japan	1967–1997	8.4	544	6	1.4	0.53–3.12	1.1	4570^a^
Oriuchi	2003	Japan	1965–1998	9.9	276	11	N.D^b^	N.D^b^	4.0	2732^a^
Kennedy	2012	Scotland	1998–2000	3.0	1595	144	3.3	2.8–3.89	9.0	4785^a^
Lee	2017	Korea	1981–2013	8.6	2414	35	1.4	0.97–1.94	1.4	20,712
Yasukawa		Japan	1968–2015	14.6	1108	52	3.5	2.7–4.6	4.7	16,199

*SMR* standardized mortality ratio, *CI* confidence interval

^a^Person-years at risk were estimated by the author (cases × follow-up periods)

^b^N.D: no data in the publication

Regarding SMRs by disease, previous reports showed that SMRs were high for neoplasms, especially small intestinal cancer and CRC, as well as gastrointestinal diseases [13, 14, 26, 27]. As in previous studies, the present study also found high SMRs for malignant tumors, especially small intestinal cancer and CRC, as well as gastrointestinal disease and amyloidosis, and not very high SMRs for respiratory and cardiovascular disease. The SMR of all causes was high in the present study, likely because of the high SMR for CD-specific causes.

In the present study, the SMR for gastrointestinal disease was high, because the data showed that the number of intestinal resections was significantly related to the death rate (Table 5). CD induces intestinal complications over the long course of the disease, requiring repeated surgeries. Frequent surgeries and intestinal complications can result in short-bowel syndrome and IF. According to Watanabe et al., IF was observed relatively frequently at a specialized hospital, with an incidence of 3.6% at 10 years and 8.5% at 20 years after initial surgery for CD, and the mortality after diagnosis of IF was 3.7% at 5 years and 8.9% at 10 years [28]. HPN-related mortality according to reports from overseas ranges from 2 to 28% at 5 years, which is by no means low [29]. Loly et al. collected data from numerous cases and stated that the mortality of CD patients who received HPN was 10% at 5 years, and Limketkai et al. stated that mortality caused by IF was 6% at 1 year and up to 20% at 4 years [30, 31]. It could be expected that mortality due to intestinal complications, as well as short-bowel syndrome and IF caused by such complications, would be high, and the SMR for gastrointestinal disease is considered to be high, similar to previous reports.

Malignant tumors had the second highest SMR in the present study. Recently, Caini et al. from Italy conducted long-term follow-up of 231 CD patients and compared SMRs by causes of death. Their results showed that the SMR was 1.79 for all causes (95% CI 1.39–2.27), and it was very high for cancer, at 2.57 (95% CI 1.28–5.13) [32]. Furthermore, most reports have demonstrated that the SMR is higher for lower gastrointestinal cancer among malignant tumors. Reports from Western countries have shown the standardized incidence ratio (SIR) to be 2.5 (95% CI 1.3–4.7) for CRC and 33.2 (95% CI 15.9–60.9) for small-bowel cancer [33]. It has also been reported in Japan that the SIR for CRC is 2.79–5.8 [34, 35]. Higashi et al. reported that the frequency of comorbid CRC in CD from multiple facilities specialized for IBD was 3.5% (122/3454 patients), and that the 5-year survival rate of CRC was 88% in Stage I, 68% in Stage II, 71% in Stage IIIa, 25% in Stage IIIb, and 0% in Stage IV [36]. Of these cases, 91% of the diagnosed gastrointestinal cancers were advanced cancers. In addition, they claimed that comorbid CRC has increased markedly in Japan since 2008. Sugita et al. analyzed 20 CD patients with rectal and anal cancers, and they reported a mortality of 33% after 36 months [37]. As described here, the incidences of intestinal cancer and anal cancer have also increased in Japan, and mortality associated with these diseases is not low.

One of the major differences between this study and previous reports is that the SMR for amyloidosis was very high. The prevalence of comorbid secondary amyloidosis in patients with CD has been reported to be 2.5–5.3% in Japan and 0.9–5.6% in Western countries [38–42]. It is considered that, when amyloidosis develops in conjunction with CD, serious complications including kidney failure ensue, ultimately increasing mortality [42]. A recent study from the USA showed that mortality increases in the presence of secondary amyloidosis in hospitalized CD patients [41]. A report by Miyaoka from Japan stated that secondary amyloidosis does not occur frequently in CD (2.5%), but that 40% of these patients died, indicating that the prognosis is poor with secondary amyloidosis [40]. In addition, a long-term outcome report by Weterman et al. showed that 4 of 64 deaths (6.3%) were due to amyloidosis [22], indicating that this is not rare. Patients are diagnosed with amyloidosis between 12.0 and 25.0 years after the diagnosis of CD [38, 40, 43], and they die of complications such as kidney failure. Thus, death caused by amyloidosis may only be captured by performing long-term follow-up, as demonstrated in the present study.

The limitations of this study are as follows. It is very difficult to obtain long-term outcomes including mortality rates in a cohort group, because CD patients have younger disease onset, and follow-up for more than 20 years is extremely difficult due to their higher social activity and frequent changes in residence. Because we have an affiliated hospital network and added a questionnaire survey, we succeeded in gathering sufficient data related to mortality and cause of death over a long term.

Because the present study was a retrospective cohort study, disease location and behavior observed at initial examination were not significant factors associated with mortality. Furthermore, while the usefulness of anti-TNF-α agents and a decrease in the intestinal resections performed for refractory CD have been suggested recently, there was no association with the use of medications [44–47]. Nutritional therapy was not considered in the present study; it was considered difficult to elucidate the contribution of nutritional therapy to mortality since the data were old and retrospective, and the dose and duration of the therapy were not known in many of the patients. Further investigations will be necessary to clarify the associations with treatment and changes in disease type.

## Conclusion

The long-term follow-up of CD patients in the present study showed that the cumulative survival rate decreases and SMR increases ≥ 25 years after diagnosis. Among individual complications, CD-specific causes such as intestinal cancer, IF, severe (fistulous) diseases including perioperative complications, and amyloidosis developed ≥ 20 years after diagnosis, increasing the mortality. In the future, it may be possible to improve the prognosis by predicting these serious complications or by diagnosing them at an early stage.

## Abbreviations

CD: Crohn’s disease
CI: Confidence interval
CRC: Colorectal cancer
HPN: Home parenteral nutrition
HR: Hazard ratio
IBD: Inflammatory bowel disease
ICD: International Classification of Diseases
IF: Intestinal failure
SMR: Standardized mortality ratio
SIR: Standardized incidence ratio
TNF: Tumor necrosis factor
